# Conditional reprogramming: Modeling urological cancer and translation to clinics

**DOI:** 10.1002/ctm2.95

**Published:** 2020-06-05

**Authors:** Wei Liu, Lingao Ju, Songtao Cheng, Gang Wang, Kaiyu Qian, Xuefeng Liu, Yu Xiao, Xinghuan Wang

**Affiliations:** ^1^ Department of Urology Zhongnan Hospital of Wuhan University Wuhan China; ^2^ Department of Biological Repositories Zhongnan Hospital of Wuhan University Wuhan China; ^3^ Human Genetic Resources Preservation Center of Hubei Province Wuhan China; ^4^ Department of Pathology, Lombardi Comprehensive Cancer Center Georgetown University Medical Center Washington DC; ^5^ Medical Research Institute Wuhan University Wuhan China

**Keywords:** conditional reprogramming, patient‐derived model, precision medicine, urological cancer

## Abstract

Patient‐derived models, including cell models (organoids and conditionally reprogrammed cells [CRCs]) and patient‐derived xenografts, are urgently needed for both basic and translational cancer research. Conditional reprogramming (CR) technique refers to a co‐culture system of primary human normal or tumor cells with irradiated murine fibroblasts in the presence of a Rho‐associated kinase inhibitor to allow the primary cells to acquire stem cell properties and the ability to proliferate indefinitely in vitro without any exogenous gene or viral transfection. Considering its robust features, the CR technique may facilitate cancer research in many aspects. Under in vitro culturing, malignant CRCs can share certain genetic aberrations and tumor phenotypes with their parental specimens. Thus, tumor CRCs can promisingly be utilized for the study of cancer biology, the discovery of novel therapies, and the promotion of precision medicine. For normal CRCs, the characteristics of normal karyotype maintenance and lineage commitment suggest their potential in toxicity testing and regenerative medicine. In this review, we discuss the applications, limitations, and future potential of CRCs in modeling urological cancer and translation to clinics.

## Graphical abstract

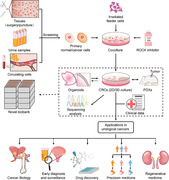

**Abbreviations**
3D: three‐dimensional
ALP: alkaline phosphatase
BCa: bladder cancer
CR: conditional reprogramming
CRCs: conditionally reprogrammed cells
CTCs: circulating tumor cells
CYPs: cytochromes p450
Dkk1: dickkopf‐related protein 1
ESCs: embryonic stem cells
iPSCs: induced pluripotent stem cells
PCa: prostate cancer
PDMs: patient‐derived models
PDX: patient‐derived xenograft
PHHs: primary human hepatocytes
RCC: renal cell carcinoma
ROCK: Rho‐associated kinase
RRP: recurrent respiratory papillomatosis
SCID: severe combined immunodeficiency
SmCC: small cell neuroendocrine carcinoma
TDCM: transwell dish culture method
VMY: VMY‐1‐103

## 1 BACKGROUND

Urological cancers consist of the cancers that occur in the prostate, urinary bladder, kidney, and other organs of the urinary system. It has been estimated that 351 050 new cases and 67 150 deaths from urological cancers will occur in the United States in 2020.^1^ Consistent with the data from the past several years, prostate cancer (PCa) still represents the most common malignancy in men in the United States. Given the considerable burden of cancer, the use of advanced techniques to understand tumor progression and treatment outcomes in urological cancer is still the ultimate goal pursued by researchers around the world.

Currently, the limited availability of cancer models is a major bottleneck that limits the progress of cancer research.^2^, ^3^, ^4^ Traditionally, cancer cell lines have extensively served as efficient models for oncology research, drug discovery, and preclinical studies.^5^, ^6^ However, the success rate is as low as 1‐10% for establishing cancer cell lines,^7^, ^8^ depending on the origin and progression of the disease.^9^ To date, despite a steady increase, the existing cell models are still insufficient to facilitate the study of rare cancers and/or specific cancer subtypes. Additionally, traditional cancer cell lines have the limitation that they cannot comprehensively recapitulate the complex heterogeneity of primary tumors,^10^ thus greatly limiting the development of basic and translational medicine. On the other hand, animal models are at the center of laboratory cancer research.^11^ They have greatly facilitated our understanding of the etiology and biology of malignancies and have proven useful for preclinical studies of new therapeutics. Generally, animal models for cancer research encompass chemical carcinogenesis models, genetically modified animals, xenograft models, syngeneic models, and others.^12^ Although these models are proposed to substitute for human beings in cancer research, some heterogeneity in reality exist between the different species. Therefore, it is not difficult to explain why the extrapolation of experimental studies into clinical practice is slow with the use of laboratory animals.^12^ To overcome these challenges, novel feasible models are urgently needed for current cancer research and translational medicine.

With the development of biotechnology in recent years, the systematic approach to generating cancer models has changed dramatically. Patient‐derived models (PDMs) may be one of the most “shining stars,” retaining consistent genetic backgrounds with their parental generations. Organoids, induced pluripotent stem cells (iPSCs), patient‐derived xenografts (PDXs), and conditionally reprogrammed cells (CRCs) that serve as PDMs have been frequently used in recent years. These models play important roles in different areas of cancer research depending on the context and technology they generate.^13^, ^14^, ^15^, ^16^, ^17^ In this review, a brief comparison of these PDMs is discussed (also in Table 1). Importantly, because the emerging conditional reprogramming (CR) technique has attracted much attention in cancer research, the current status and potential applications of CR technology in urological cancer research are comprehensively reviewed in this article.

**Highlights**

- The CRC technique enables the generation of the patient‐derived tumor and/or normal cells.
- Tumor CRCs can share certain genetic aberrations and tumor heterogeneity with their parental tumors.
- CRCs may serve as a promising platform to facilitate cancer research in many aspects, including transforming biobanking repositories.

**TABLE 1:** Comparisons between patient‐derived models: induced pluripotent stem cells (iPSCs), organoids, patient‐derived xenografts (PDXs), and conditionally reprogrammed cells (CRCs)

Models	Advantages	Shortcomings
iPSCs	Pluripotent differentiation Can combine with gene editing and 3D organoids	Slow and inefficient procedure Difficult to reprogram cancer cells Safety issues
Organoids	3D culturing Can generate both healthy and tumor organoids Maintained genetic aberrations in tumor organoids	Dependent on stem cells Long manipulation line Overgrowth of nonmalignant cells
PDXs	In vivo model Direct engraftment from human tumor Preserved tumor heterogeneity and lineage hierarchy Tumor‐stromal interactions	Expensive Long manipulation line (6 months to 2 years) Varied engraftment rate (10‐90%) Low‐throughput drug screening Only tumor models
CRCs	Extensive specimen sources Paired normal and tumor cells culturing Cost saving and rapid expansion (1‐10 days) Can maintain original karyotype and tumor heterogeneity High‐throughput drug screening	Contamination with feeder cells Overgrowth of benign cells Lack of stromal components

## 2 PATIENT‐DERIVED CANCER MODELS

### 2.1 Induced pluripotent stem cells

The human pluripotent stem cell‐derived procedures have provided new avenues for biomedical research.^18^ Pluripotent stem cells, such as iPSCs and embryonic stem cells (ESCs), retain the ability to differentiate to all functional cells of the body.^19^ However, due to technical and ethical issues, the medical use of somatic cell nuclear transfer and ESCs is hindered.^18^, ^20^ Thus, iPSCs emerged as a robust technique with great potential for disease modeling. Regarding the technique, iPSCs are generated from somatic cells through the transient exogenous expression of a set of transcription factors (Oct‐4, Sox‐2, Klf‐4, and c‐Myc, collectively termed “OSKM” factors in the original protocol).^21^ Exogenous expression of these transcription factors induces massive epigenetic remodeling and ultimately leads to the activation of an endogenous network of pluripotency regulators.^22^, ^23^ In principle, the established iPSCs are thought to be indefinitely maintained in culture and can be cryopreserved and expanded without loss of their genetic and phenotypic properties.^23^, ^24^, ^25^, ^26^ To date, in addition to reprogramming normal cells, many iPSCs from cancer cells have been successfully generated.^23^, ^27^, ^28^, ^29^, ^30^ In the field of urology, the iPSC lines can be derived from somatic cells from patients with hereditary renal cell carcinoma (RCC)^31^ and from primary dermal fibroblasts from patients with Von Hippel‐Lindau syndrome (a familial cancer syndrome).^32^ Furthermore, it has been reported that cancer cells can even be reprogrammed into normal functioning cells.^33^, ^34^

Currently, the combination of the iPSC platform with gene editing and the emerging three‐dimensional (3D) organoid technology can make human iPSC an even more useful technique.^16^, ^17^ Even so, iPSC technology does have several limitations that remain to be overcome. Given the complexity of the reprogramming process, this technique is an intrinsically slow and less efficient procedure, where less than 3% of the initiating cells can be reprogrammed into iPSCs using the original protocol.^35^ Nevertheless, studies have reported that higher reprogramming efficiency of iPSC can be achieved by replacing part of the “OSKM” inducers^36^, ^37^, ^38^ or adding certain small molecule compounds.^39^, ^40^ The safety issue is another concern associated with the exogenous import of transcription factors, which may activate unexpected oncogenic pathways.^41^, ^42^ In addition, human iPSCs tend to differentiate into cells with immature embryonic or fetal identity rather than a fully mature adult state.^16^, ^23^ All these issues need further investigation.

### 2.2 Organoids

Organoids are 3D cell structures derived from neonatal, pluripotent, or adult stem cells, which spontaneously self‐organize and underdo a degree of differentiation to give rise to functional cell types and have the ability to assume certain functions of the relevant organs.^43^, ^44^, ^45^, ^46^, ^47^ Initially, Sato et al developed a method that could generate continuously expanding, self‐organizing intestinal organoids by culturing them in a Matrigel protein matrix.^48^ In 2011, healthy organoids derived from patients were successfully cultured.^49^ Since then, many types of organoids from different human tissues, normal and/or neoplastic, have been unprecedentedly developed.^50^, ^51^, ^52^, ^53^, ^54^

In the field of urological oncology, 3D prostate organoids have been successfully established from human healthy prostate cells, metastatic lesions, and circulating tumor cells (CTCs).^50^, ^55^ Organoids derived from healthy tissues contained the differentiated basal and luminal cell types, whereas those derived from PCa materials shared mutational landscapes with that of the parental tumors.^50^, ^55^ Bladder cancer (BCa) organoids have also been successfully established by using patient resection samples ranging from nonmuscle invasive diseases to high‐grade muscle invasive cancers.^56^, ^57^ Based on immunohistochemistry and sequencing analysis, these resulting BCa organoid lines contained both basal and luminal subtypes, and common mutations, such as *TP53* and *FGFR3*, were also detected.^57^ In addition, Lee et al have shown that organoids and orthotopic xenografts could be interconverted with high efficiency, which indicated that these models could be employed to validate drug responses, test agent toxicity, and further develop novel treatment strategies.^56^ In kidney cancer, Batchelder et al successfully established RCC organoids via a 3D cell‐scaffold system. As a result, the gene expression profiles of these cells were consistently maintained in 3D cultures for up to 21 days.^58^ Collectively, the advantage of organoid culturing lies mainly in the ability to generate both tumor and normal cell lines from the same patient, and it supports 3D culture, which can mimic cell‐cell and cell‐matrix interactions. Increasing evidence suggests that patient‐derived organoids are genetically stable and can faithfully recapitulate the main features of a patient's disease, including genetic heterogeneity and response to therapeutics.^59^ Moreover, the potential use of organoids is not limited to modeling neoplastic and nonneoplastic diseases, but can also be used in regenerative medicine.^60^, ^61^

Despite its promise, the limitations and technical challenges for organoids cannot be ignored. Because of the overgrowth of nonmalignant cells, the success rate of generating organoids from malignancies is as low as 15‐20%.^13^ Moreover, organoids are more feasible for low‐ rather than high‐throughput drug screening.^62^, ^63^

### 2.3 Patient‐derived xenografts

PDXs are generated by engraftment of human tumor fragments into immunocompromised mice.^14^, ^15^, ^64^ Because a PDX model retains the properties of the primary patient tumor, including gene expression profiles and drug responses, it has become the most reliable in vivo human cancer model and is now being widely used in cancer research.^15^, ^65^ Within the field of urology, several types of xenografts can imitate the major characteristics of PCa patients, such as hormone dependence/independence and the ability to induce castration‐resistant PCa in mice through androgen ablation and other methods.^66^ RCC is particularly well suited for the establishment of PDXs that recapitulate the clinical situation.^14^ RCCs can usually provide abundant tumor materials when progressing to locally advanced diseases. Most RCC surgeries are rarely performed after medical treatment; therefore, the molecular genetics of a tumor is unlikely to be affected by the medication.^14^ RCCs can also be implanted under the renal capsule, which is a privileged site for tumor survival and growth to generate orthotopic xenograft models.^67^, ^68^ All these features enable RCCs to become ideal tissue sources for generating available PDXs. Currently, RCC PDX models are commonly utilized for testing drug responses and exploring mechanisms of resistance to agents, especially in targeted molecular therapies.^69^, ^70^, ^71^ For urothelial carcinoma, the success rate of establishing PDX tumors of high‑grade disease is higher than that of establishing PDXs from RCC or PCa.^14^ To date, more than 70 urothelial carcinoma PDX models have been reported in the literature, although few upper tract urothelial carcinoma‐derived PDX lines have been established.^14^, ^72^ At present, the established urological PDX models are increasingly utilized for biomarker discovery, the study of tumor differentiation, and genomic profiling for novel drug development.^73^, ^74^

Despite their benefits, PDX tumor models still have some limitations. First, the establishment of PDXs is relatively expensive and time‐consuming (6‐24 months), and the success rate varies (10‐90%) by tumor origins and disease characteristics.^15^, ^75^ Another limitation of PDXs is the rapid loss of human stromal components, which are replaced by the murine microenvironment during engraftment.^76^ The new murine stroma may lead to changes in the paracrine regulation of the tumor as well as in physical properties such as interstitial pressure, which may limit the study of drugs targeting this tumor compartment.^77^ Additionally, immunodeficient hosts are essential for the establishment of PDX models. Thus, the PDX models generally lack principal immune cells and cannot fully recapitulate the response of the human immune system to the tumors and the tested drugs. To overcome this limitation, mice with a reconstituted human immune system, called humanized mice, have been established to offer a unique platform for examining human immune responses to the relevant tumors and for evaluating immune therapies.^15^, ^78^ In summary, at least so far, the limitations mentioned above have hindered PDXs to provide practical references for clinical decision‐making. There remain some key issues to be resolved to make this platform more informative.

### 2.4 Conditionally reprogrammed cells

**FIGURE 1:**
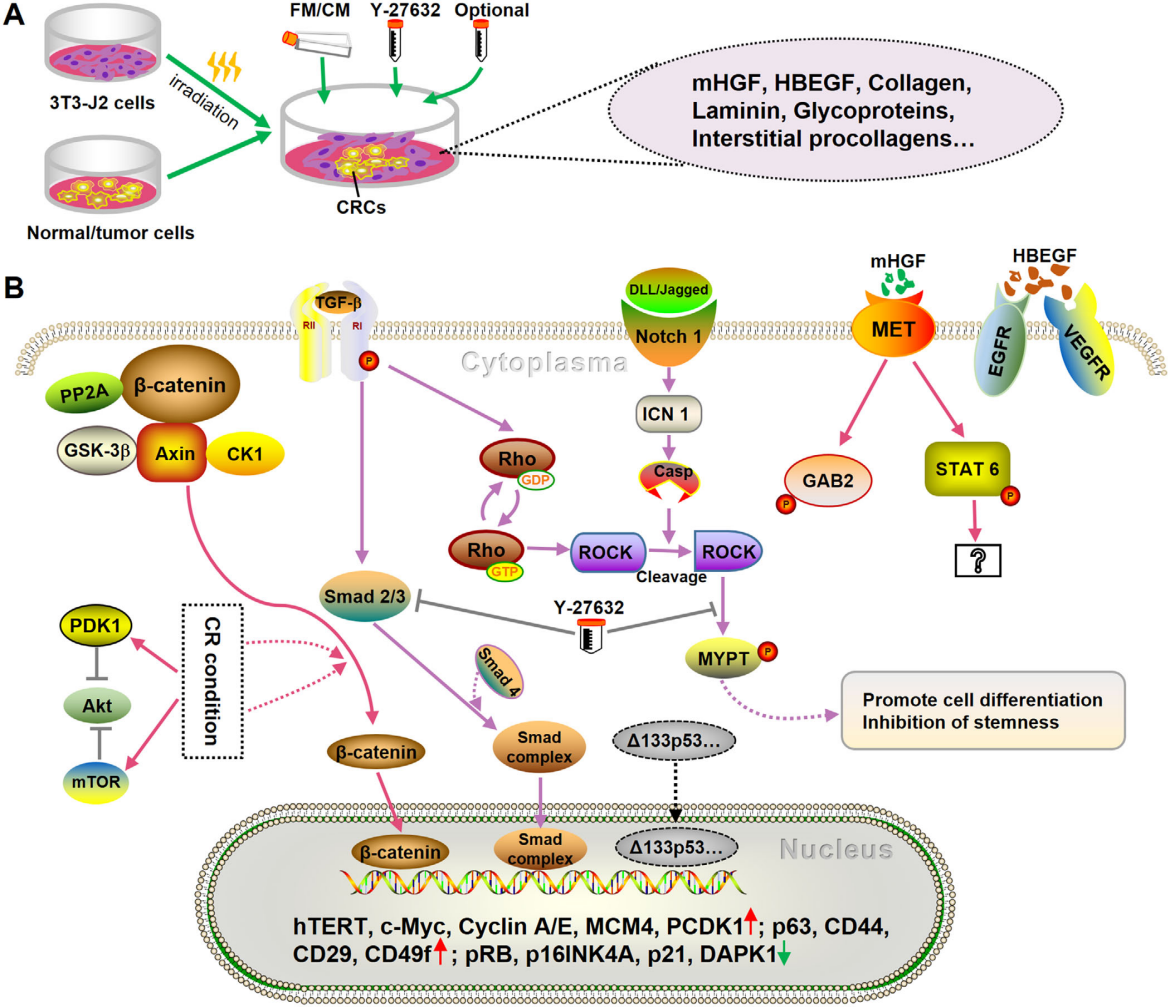
The conditional reprogramming (CR) culture system and the potential molecular mechanisms of CR. A, The CR co‐cultures patient‐derived primary normal or tumor cells with irradiated Swiss‐3T3‐J2 mouse fibroblasts (served as feeder cells) in the medium containing F medium (FM)/conditioned medium (CM), Y‐27632 (Rho‐associated kinase [ROCK] inhibitor), and optional components (ie, collagen solution, poly‐l‐ornithine solution, B‐27, R‐spondin‐1, N‐2 supplement, etc; the optional components are adjusted to specific cultures).^9^ The J2 feeder cells can produce diffusible factors (eg, murine hepatocyte growth factor [mHGF] and heparin‐binding epidermal growth factor [HBEGF]) and extracellular matrix (eg, collagen, laminin, glycoproteins, interstitial procollagens, etc) which may promote the proliferation, growth, and attachment of the cultured conditionally reprogrammed cells (CRCs).^91^, ^92^ B, Potential signaling pathways involved in the CR process. Under CR condition, β‐catenin is activated in a protein phosphatase 2A (PP2A)‐dependent manner (noncanonical β‐catenin pathway). The activated β‐catenin, upon nuclear translocation, stimulates an increase in transcripts such as Axin2, CD44, and c‐Myc that are important for maintaining the adult stem‐like state of CRCs.^94^ Meanwhile, the mTOR signaling is activated in CRCs, which is found to significantly reduce Akt activity.^94^ Treated with ROCK inhibitor (Y‐27632), the TGF‐β/SMAD pathway^95^ and noncanonical NOTCH signaling can be blocked.^96^ As a result, the differentiation of CRCs is inhibited, whereas the stemness of them is maintained.^96^ Moreover, the J2 feeder cells could secrete diffusible factors such as mHGF and HBEGF that may activate MET, EGFR, and VEGFR signaling.^92^, ^93^ Regarding protein expression, the cultured CRCs express an elevated level of hTERT,^97^ cell cycle‐related proteins (Cyclin A/E, MCM4, and PCDK1),^92^ and stem cell markers (p63, CD44, CD29, and CD49f),^99^ whereas they express inactivated pRB, p16INK4A, p21, and DAPK1.^99^, ^100^, ^101^ As a result, the potential mechanisms of CR technology may rely on the interaction of these genes and signals to promote cell proliferation, inhibit apoptosis and differentiation, and maintain unlimited proliferative capacity,^102^ thereby allowing the culture of patient‐derived primary cells. It is noteworthy that the current exploration of CR mechanisms is very limited, and almost all are based on the scenario of normal epithelial cell culture. More in‐depth investigations are needed in the future.

**FIGURE 2:**
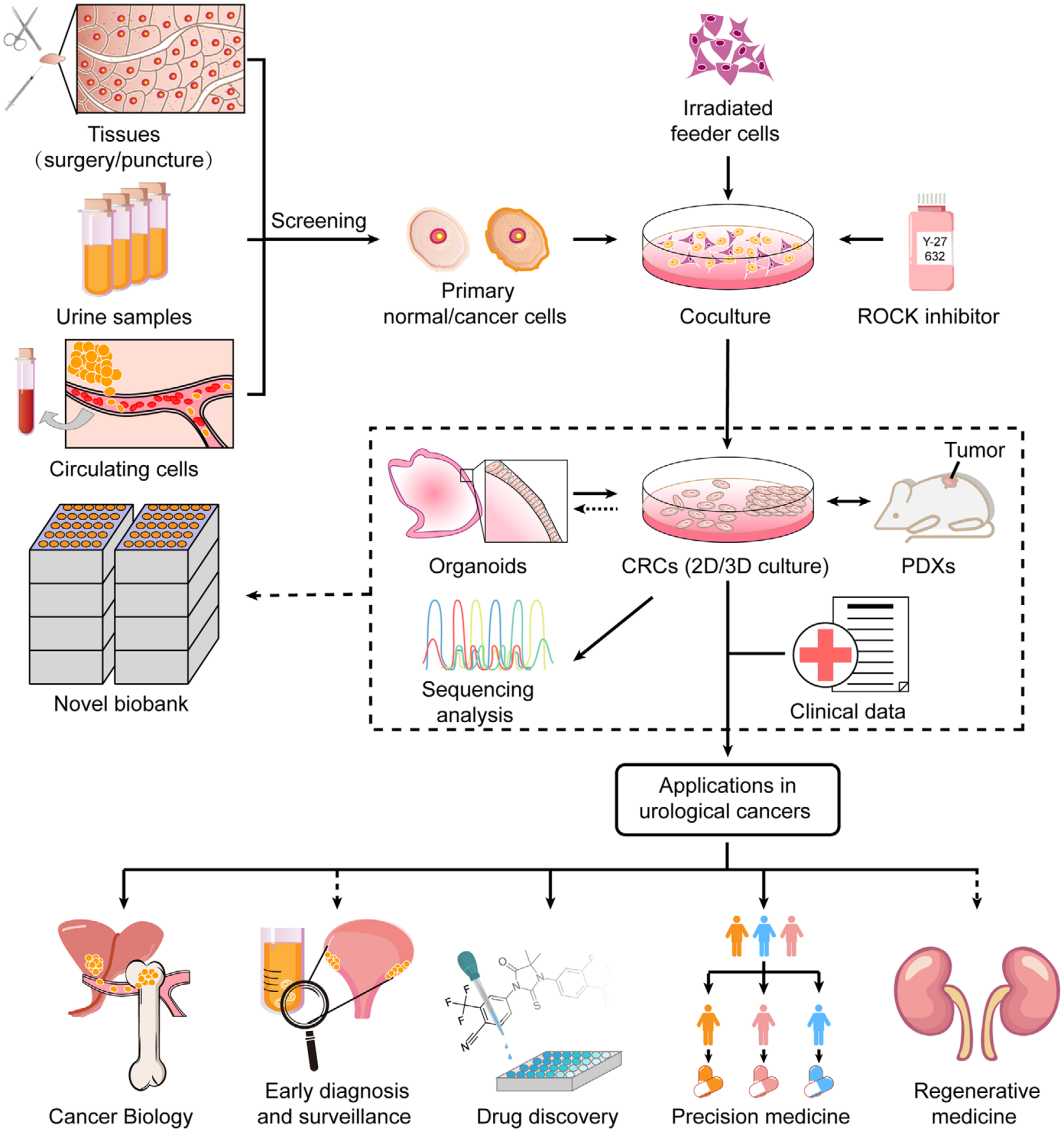
Workflow of the conditional reprogramming (CR) method for current application and future potential in urological cancer research. Briefly, specimens are collected from surgical excisions, core biopsies, or liquid biopsies (urine or blood samples) from patients with organ‐confined, metastatic, or even any stage of tumors. The samples are thoroughly evaluated by a pathologist to identify the composition (ie, to ensure its normal/tumor status). Then, the samples are dispersed into single cells by enzymatic digestion and plated in medium containing irradiated J2 feeder cells and Y‐27632 (Rho‐associated kinase [ROCK] inhibitor). The established conditionally reprogrammed cell (CRC) cultures should be validated by sequencing analysis. The CRCs can be used for various applications (not only in urological cancers), including the study of cancer biology, drug discovery, precision medicine, and promising for regenerative medicine and early diagnosis and surveillance of malignancies. Additionally, the CRCs can be used to establish patient‐derived xenografts (PDXs), and the CRCs can also generate cell cultures from PDXs and organoids. All these patient‐derived models in combination with clinical patient data provide great opportunities to create novel biobanks.

**FIGURE 3:**
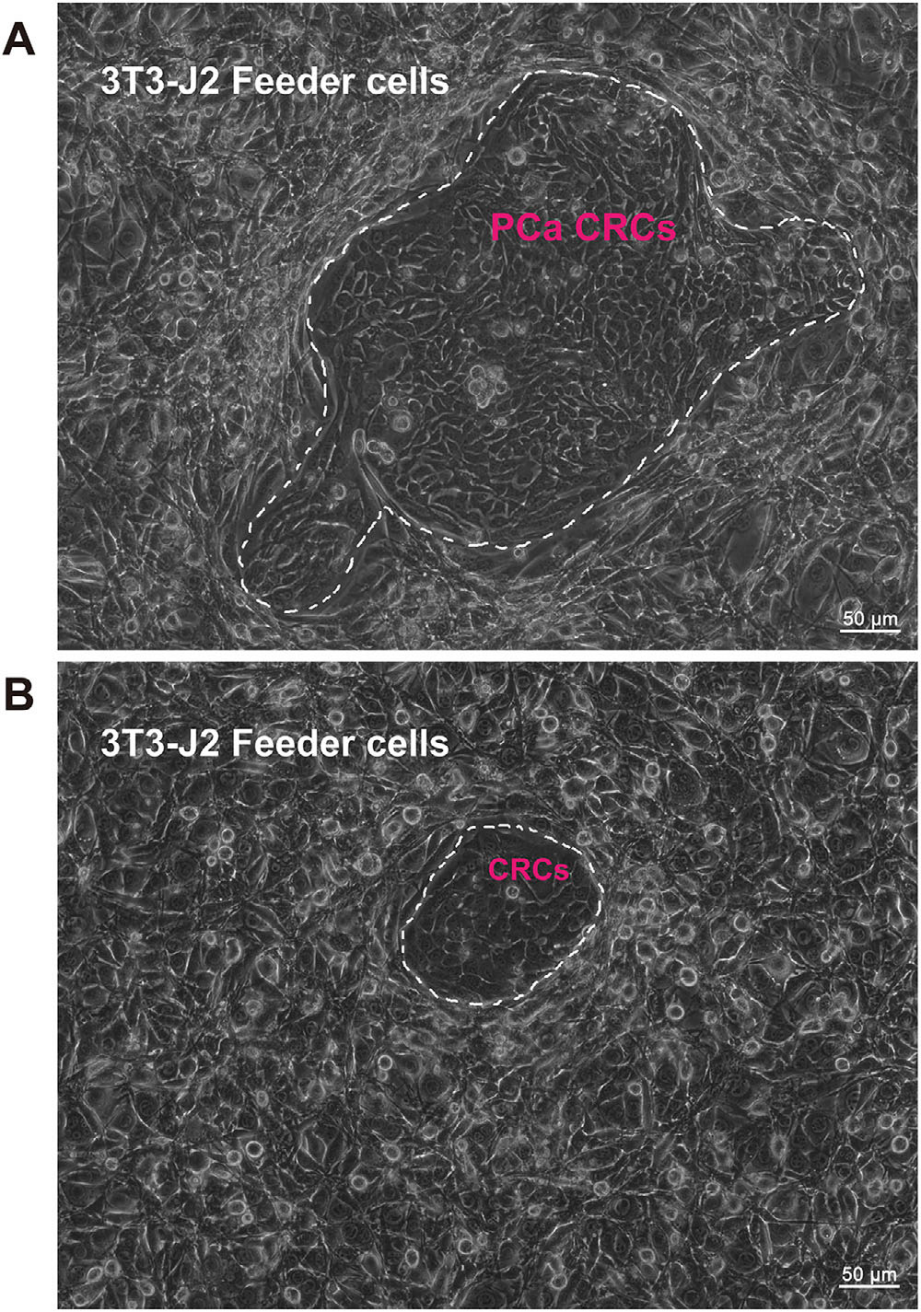
In vitro culture of conditionally reprogrammed cells (CRCs) from prostate cancer patients. A, Under light microscope, the established prostate cancer CRCs (inside the dashed coil and labeled in red) formed tight colonies and were surrounded by 3T3‐J2 feeder cells (outside the dashed coil and labeled in white). The primary prostate cancer cells were isolated from surgically resected tissues of a patient with prostate cancer disease (pT3N0Mx, Gleason score: 4 + 5). B, A light microscope image of CRCs and 3T3‐J2 feeder cells. The CRC culture was established based on the cells isolated from the urine sample of a patient diagnosed with prostate cancer (T3bN0M0, Gleason score: 4 + 5). It should be noted that the derivation of the established CRCs (whether it was prostate cancer‐derived or normal epithelium‐derived) requires further validation. Scale bars, 50 µm.

CR, which is emerging as a novel platform to generate human primary tumor and/or normal cells, has attracted great attention in recent years. Using the CR technique, normal and tumor cells can be rapidly converted to a stem‐like state, in which the culturing cells are highly proliferative and can retain their original karyotypes.^62^ The detailed protocol of CR culturing has been described in previous studies.^9^, ^62^ Briefly, human tissue specimens are obtained from core biopsies, surgical excisions, or PDX tissues. The specimens are thoroughly assessed by a pathologist to evaluate the composition (ie, to ensure its normal/tumor status) using histological methods. Then, the samples are dispersed into single cells by enzymatic digestion and plated in medium containing Y‐27632 (Rho‐associated kinase [ROCK] inhibitor) and irradiated 3T3‐J2 mouse fibroblasts (served as feeder cells) (Figures 1A and 2). Under CR condition, the epithelial cells can form colonies within a few days (see an exemplar of cultured PCa‐derived CRCs in Figure 3). Subsequently, a sequencing analysis should be performed on both the CRCs and their parental tissues to validate the derivation of the resultant cells.^9^, ^62^ Interestingly, the induction of CRCs is reversible; thus, the removal of Y‐27632 and feeder cells allows the CRCs to differentiate normally, which is why this technique was named “conditional reprogramming.” For example, when CRCs from the tracheal epithelium or ectocervical epithelium are placed in an air‐liquid interface culture system, the tracheal cells form a ciliated airway epithelium, whereas the cervical cells form a stratified squamous epithelium.^79^

Currently, many types of CRCs have been successfully established from neoplastic and/or normal epithelial tissues.^80^, ^81^, ^82^, ^83^, ^84^, ^85^ In addition to the generation of primary cancer/normal cell lines, CR can be used to establish organoids,^62^ xenografts,^79^, ^86^ and PDX cell lines,^87^ and CR can also generate cell cultures from PDXs and organoids.^88^, ^89^, ^90^ For example, the matched normal and tumor organoids could be established from the tongue cancer CRCs using 3D culturing.^62^ As reported, the resultant organoid cultures demonstrated their corresponding morphological and proliferative characteristics and the analysis of specific molecular markers confirmed the squamous epithelial origin of the cultures.^62^ In turn, the PCa CRCs have been reported to derive from their corresponding organoid lines with karyotype commitment.^90^ Thus, the translation between these PDMs suggests that these platforms may work synergistically to facilitate cancer research using primary patient‐derived cells.

To date, despite its robust feature, the mechanisms of CR remain to be well interpreted. Under CR condition, the 3T3‐J2 feeder cells can produce diffusible factors (eg, murine hepatocyte growth factor [mHGF] and heparin‐binding epidermal growth factor [HBEGF]) and extracellular matrix, which may promote the proliferation, growth, and attachment of the cultured CRCs.^91^, ^92^ Several signaling pathways have also been reported to be involved in the CR process (more details in Figure 1B).^92^, ^93^, ^94^, ^95^, ^96^ In the co‐culture system, the mTOR signaling and noncanonical β‐catenin pathway were found activated,^94^ whereas the TGF‐β/SMAD pathway^95^ and noncanonical NOTCH signaling could be blocked.^96^ In addition, the CR process involves changes in the expression of many proteins. Studies showed that the cultured CRCs expressed an elevated level of hTERT,^97^ Δ133p53α,^98^ cell cycle‐related proteins (Cyclin A/E, MCM4, and PCDK1),^92^ and stem cell markers (p63, CD44, CD29, and CD49f),^99^ whereas they expressed low level of pRB, p16INK4A, p21, and DAPK1.^99^, ^100^, ^101^ As a result, the potential mechanisms of CR may rely on the interaction of these genes and signals to promote cell proliferation, inhibit differentiation and apoptosis, and maintain unlimited proliferative capacity,^102^ thereby allowing the culture of patient‐derived primary cells. However, the current exploration of CR mechanisms is very limited, and almost all are based on the scenario of normal epithelial cell cultures. Therefore, more in‐depth investigations are needed in the future.

In summary, CR culturing can obtain large numbers of human primary cells in a short time without any exogenous gene transduction and can largely preserve cell lineage commitment and retain cell heterogeneity present in parental tissues.^62^, ^103^ Thus, CR can promisingly be used for various applications, including the study of cancer biology, high‐throughput drug screening, personalized treatment, and promising for regenerative medicine, early diagnosis, and surveillance of malignancies. In this review, we discuss the current applications (Table 2) and the potential of the CR technique in urological cancer research as follows (Figure 2).

**TABLE 2:** Studies of conditionally reprogrammed cells (CRCs) applied in urological cancer research

Diseases	Sample collections	Investigations	References
Human PCa (Gleason 6 and 8)	Radical prostatectomy specimens	Multidimensional culturing; CRCs phenotypic profiling; analysis of lineage commitment and effect of culture conditions on functional protein expression.	^111^
Human metastatic PCa	Lymph node and bone samples	To develop an ex vivo 3D bone model and investigate metastatic PCa interactions with osteocytes.	^90^
Human PCa (T3b, Gleason 7)	Prostatectomy specimens	CRCs’ viability and phenotype profiling; combination with PDX method; karyotype and exome sequence analysis and drug testing.	^110^
Human PCa (pT3aN0M0‐cT4N1M1)	Surgical resections and needle biopsies	CRCs phenotypic profiling, genetic aberration profiling and drug sensitivity testing.	^124^
Human PCa (Gleason 7)	Radical prostatectomy specimens	A novel drug sensitivity testing.	^125^
Human PCa (Gleason 7)	Radical prostatectomy specimens	To investigate the role of p53 gene in VMY‐induced prostate cancer cell death.	^126^
Human BCa (low grade and high grade)	Urine samples and surgical resections	To establish BCa CRCs from tumor tissues and urine samples and applied the cultures for whole exome sequencing and drug testing.	^117^
Human BCa (four pTaN0‐T4N1 high‐grade urothelial carcinoma; one pT4aN1 SmCC; one pT2bN1 adenocarcinoma)	Cystectomy or transurethral specimens	To investigate the suitability of tumor‐derived CRCs for the characterization of BCa properties and their feasibility for personalized drug sensitivity screening.	^83^
Human BCa (pT2NxMx/pT2aN2Mx/pT4N0Mx)	PDX‐derived tumor samples	To establish PDX‐derived tumor CRCs and determine whether PDXs and CRCs of the same cancer origin maintain the biological fidelity.	^89^
Human RCC (pT3N0‐pT4NxM1)	Nephrectomy specimens	To establish CRCs from different tumor regions, verify their clonal relationships to each other and to parental tumor tissues and conduct comprehensive drug sensitivity testing.	^120^

## 3 APPLICATIONS OF CR IN UROLOGICAL CANCERS

### 3.1 Cancer biology

Tumorigenesis is a hot topic in the field of cancer biology. Generally, PCa displays a strongly luminal phenotype^104^; thus, by inference, it should stem from luminal cells. However, many previous studies have suggested that PCa may originate from basal cells.^105^, ^106^, ^107^ As a result, the cell of origin of PCa remains controversial.^104^, ^108^, ^109^

By CR technology, Timofeeva et al established paired tumor and normal cultures from a patient's prostatectomy specimen, and they injected CRCs subcutaneously into adult male severe combined immunodeficiency (SCID) mice to observe the genetic maintenance between the culturing models and the parental tumors.^110^ Gratifyingly, these patient‐derived CRCs proliferate indefinitely in vitro and maintain stable karyotypes. More importantly, only tumor‐derived CRCs grew into tumors in SCID mice, suggesting that a critical tumor phenotype is maintained. The results of flow cytometry and polymerase chain reaction analysis showed that both normal and tumor CRCs expressed an elevated level of basal cell markers (which suggested transit‐amplifying phenotypes), whereas a decreased level of luminal markers. However, after the injection of tumor‐derived CRCs into SCID mice, the expression of luminal markers increased remarkably; on the contrary, the level of basal cell markers decreased dramatically. This conversion may suggest the origin of PCa. However, the influence by the presence of components in the CR culturing system, such as feeder cells and ROCK inhibitor, cannot be ruled out. In the next year, Tricoli et al developed a novel filter‐based multidimensional culture platform, that is, the transwell‐dish culture method (TDCM),^111^ based on the two‐dimensional CR culturing system. The TDCM could enable the growth and stratification of the tumor and normal CRCs resembling the prostate epithelium. Interestingly, when cultured in TDCM, the CRCs adopted a more differentiated status and concomitant suppression of stem‐ and transient amplifying‐like phenotypes, which were observed in conventional CR culturing.^111^ These two studies demonstrated the effect of different culture conditions on the phenotypic expression of PCa cells and suggested that multidimensional culture models may be more appropriate for studying cancer biology.

With advanced PCa, more than 80% of patients progress to bone metastases and these patients usually have a high level of morbidity, with a median survival of only 40 months.^112^ Bone metastasis is a complex disease involving synergistic interactions among tumor cells, osteoclasts, osteoblasts, and mineralized bone matrix.^113^ To date, the exact mechanism of bone metastasis has not been well elucidated. In 2018, Choudhary and colleagues established an engineered bone tissue model integrated by 3D‐networked human osteocytes with primary PCa CRCs.^90^ It was noteworthy that the established CRCs were derived from the PCa organoid lines that were generated from retroperitoneal lymph nodes of a PCa patient. The established CRCs showed consistency with the original PCa organoids by karyotyping and basic molecular analyses.^55^, ^90^ In the engineered tissue without the introduction of PCa CRCs, the osteocytes were well spread out, with dendrites protruding to neighboring cells and the endosteal layer was intact, whereas once PCa CRCs were introduced, the endosteal surface was adhered by PCa cells and the 3D tissues were compromised. Sclerostin and dickkopf‐related protein 1 (Dkk1), inhibitors of Wnt signaling, and regulators in bone metastases^114^ were used to interrogate the role of osteocytes in PCa cell‐induced bone remodeling. The results showed that sclerostin was widely expressed in osteocytes in the 3D tissues without PCa CRCs, whereas a sharp decrease in sclerostin expression was detected when osteocytes were co‐cultured with PCa CRCs. The expression profiles of Dkk‐1 showed the opposite changes. Alkaline phosphatase (ALP), an indicator of osteoblastic activity, was then tested to examine the osteoblastic characteristics of PCa bone metastasis. The results exhibited a significant increase in ALP and concomitant mineralization once PCa CRCs were added to the 3D culturing model. In addition, fibroblast growth factor 23 (FGF23), which is expressed by mature osteocytes and acts as an emerging target in bone metastasis, was observed as highly expressed by osteocytes as PCa CRCs were introduced into the 3D bone tissue. However, when PCa CRCs and osteocytes were co‐cultured in a traditional two‐dimensional culturing system, those key expressional changes could not be recaptured in osteocytes, suggesting that the engineered 3D model was an ideal system for modeling PCa and bone interactions and could be utilized for further studies.^90^ In renal and urothelial carcinomas, the corresponding multidimensional (CRC‐based) models remained to be developed for cancer biology research.

CR technology, which enables the culturing of patient‐derived normal or tumor cells and can integrate with other advanced models such as PDXs and 3D culturing systems, has the potential as a tool to investigate the basic biology of malignancies, metastatic diseases, and other disorders.

### 3.2 Noninvasive diagnosis and surveillance

In recent years, liquid biopsy has received much attention for its role in providing cancer diagnosis and surveillance in a noninvasive or minimally invasive way. Many circulating molecules, including cell‐free DNA, CTCs, circulating RNAs (miRNAs/lncRNAs/mRNAs), and cell‐free proteins, have emerged as noninvasive biomarkers for different malignancies, especially for urological cancers.^115^ Specific to urothelial carcinoma, however, despite the constant discovery of noninvasive markers based on urine and peripheral blood, urine cytology and endoscopy with biopsy currently remain the gold standard for diagnosis. This is mainly due to the fact that these two examinations can provide visual and morphological information for the early diagnosis of urinary tumors, and the subsequent pathological data can further provide an effective reference for tumor staging and grading. However, these two examinations, though robust, have inherent limitations. Urine cytology is a specific tool but is poorly sensitive for low‐grade tumors. For BCa, cystoscopy and biopsy, in spite of their high diagnostic efficacy, are invasive and cannot reliably detect small and flat tumors.^116^ Therefore, novel noninvasive diagnostic tools with high sensitivity and specificity remain to be developed. The CR technique allows for isolation and culturing of the patient‐derived tumor and normal cells without changes in karyotype, indicating that if tumor and/or normal urothelial cells in urine can be successfully isolated and cultured in vitro, CR may contribute to the diagnosis of urothelial carcinomas in a noninvasive way. Currently, Jiang and colleagues have successfully established BCa cells from patients’ urine samples by the CR technique.^117^ The overall success rate for the establishment of urine CRCs exceeded 80%, of which high‐grade BCa was 85.4% and low‐grade BCa was 75.0%. The sequencing analysis validated that these urine CRCs could retain genetic landscapes of the original tumors. In subsequent analysis, the urine CRCs were utilized for drug sensitivity tests compared with clinical responses. The results suggested a clinical consistence in drug testing using urine CRCs.

In practice, urine samples can be obtained at any time before and after treatment, which provides convenience for obtaining real‐time pathological conditions. Thus, the CR technique may have the potential to screen heterotypic cells in the culturing system and further combine with pathology to facilitate the diagnosis and even grading of urothelial carcinomas. Moreover, this technique may detect recurrences earlier and predict responses to chemotherapies or immunotherapies. Nevertheless, all these scenarios remain to be validated.

### 3.3 Precision medicine

In clinical practice, drug resistance, nonresponse to medications, and a high rate of side effects are common stumbling blocks for patient treatment. To address these issues, precision medicine has been recommended to provide patients with the optimal tailored treatment, rather than a “one‐size‐fits‐all” treatment modality.^115^, ^118^ Usually, the lack of appropriate ex vivo models is a major obstacle to the identification of biomarkers to predict the response and clinical benefit of treatment.^13^ To solve this problem, CR technology may provide a good choice.

In 2012, *The New England Journal of Medicine* published a study that explored the use of CRCs to identify therapy for recurrent respiratory papillomatosis (RRP).^119^ In this case, a 24‐year‐old RRP patient had undergone more than 350 laryngeal ablation surgeries and taken several chemotherapies to control viral‐induced tumors, but all ended up ineffective. To control the chemoresistant and progressive disease, the CR technique was approved for culturing paired normal and tumor cells from the patient for drug screening. As a result, the researchers discovered different sizes of mutant HPV‐11 genomes in the laryngeal and lung tumor CRCs, respectively, and vorinostat was identified as an effective agent. Surprisingly, after a 3‐month vorinostat treatment, the tumor sizes had stabilized.^119^ This case suggests that the CR technique has great potential to facilitate precision medicine, especially in individualized treatment. In BCa, Kettunen et al used CRCs to explore their feasibility for personalized drug screening.^83^ Initially, they established CRCs from six BCa tumors of different stages and histologies. Four CRCs were successfully propagated for genetic and protein expression profiling and compared with their parental tumors. Two out of four CRCs (urothelial carcinoma and small cell neuroendocrine carcinoma [SmCC]) corresponded well to the parental tumors. Then these two cultures were used to conduct drug sensitivity screening to identify potential drugs for the respective tumors. The results demonstrated that these two CRCs were both sensitive to conventional agents (eg, taxanes, proteasome, and inhibitors of topoisomerase) and standard chemotherapy drugs (eg, cisplatin and gemcitabine) for BCa patients.^83^ In addition, the SmCC cells were unexpectedly found to be highly responsive to statins such as atorvastatin and pitavastatin, implying that statins might be a promising cost‐effective candidate for further investigation. Saeed and colleagues established multiple CRCs from different tumor regions of four RCC patients and verified their clonal relationship to each other and the parental tumors by sequencing analysis.^120^ Subsequently, comprehensive drug testing was conducted on all CRC clones. The results demonstrated that the CRCs retained many cancer‐specific copy number alterations and somatic mutations found in the original tumor tissues. The comprehensive drug testing highlighted the sensitivity in the CRCs to conventional RCC drugs, such as temsirolimus (an mTOR‐inhibitor), and novel sensitive agents were also discovered.^120^ Individually, distinct response profiles were observed among CRCs derived from different regions (primary tumor, invasive vena cava, and adrenal metastasis) in a patient's tumor tissues, suggesting that precision medicine for cancer patients should focus on not only individual treatment but also the treatment taking intratumor heterogeneity into account. Today, apart from urological cancers, the established CRCs have been utilized for comprehensive drug sensitivity testing for patients diagnosed with breast cancer, lung cancer, and salivary gland cancer.^121^, ^122^, ^123^ The CR technology is a feasible platform for personalized drug sensitivity testing and may add to the approaches to develop individualized treatment strategies.

As a prospect, the CR technique may greatly facilitate precision medicine in urological cancers in the following aspects: (a) precision diagnosis and surveillance, especially in a noninvasive manner; (b) sensitive drug screening for individual treatment taking inter‐ and intratumor heterogeneity into account; (c) development of combination regimens; and (d) response monitoring and real‐time adjustment.

### 3.4 Drug discovery and toxicity testing

The primary purpose of preclinical therapeutic efficacy testing is to predict whether a particular compound will be successful in clinical use.^12^ The CR technology can efficiently propagate primary cells without changing genetic profiles; thus, it can serve as a high‐throughput platform to discover novel agents and screen the most sensitive agents for further studies. For example, using CRC cultures, Saeed et al conducted a high‐throughput drug testing of 306 emerging and validated anticancer drugs.^124^ They identified several potential agents and combination regimens for the CRCs from a patient with castration‐resistant PCa. Among them, the Bcl‐2 family inhibitor navitoclax, which is being tested in clinical trials, proved to be a potent drug. Pollock and colleagues explored the anticancer effect of strigolactone analogues, a novel class of plant hormones, in matched primary normal and PCa CRCs.^125^ The results showed that strigolactone analogues could specifically induce cell cycle arrest and apoptosis, whereas they had little effect on the survival and growth of normal cells. Therefore, strigolactone analogues is a promising candidate for anticancer treatment in PCa. Additionally, Ringer et al found that VMY‐1‐103 (VMY), a CDK inhibitor, could exert its cytotoxic effect on PCa CRCs through p53‐dependent autophagy, which provided implications for the clinical study of VMY.^126^ In urothelial carcinoma, statins have been shown to be effective on SmCC‐derived CRCs of BCa.^83^ Apart from urological cancers, CRCs have been used in other cancer types for high‐throughput drug screening and many novel agents have been identified. Alkhilaiwi et al identified panobinostat, dinaciclib, and forskolin as potential therapies for RRP patients by 3D CR culturing and high‐throughput drug screening.^80^ Kim and colleagues found a synthetic lethal interaction of an anticancer candidate IDF‐11774 with ATP6V0C in CR colorectal cancer cells with a low level of Bcl‐2 expression, which indicated a combination regimen for further investigation.^127^ Moreover, screening of patient‐derived malignant CRCs identified ERCC3‐Myc interaction as a target in pancreatic cancer.^128^ In the future, more novel potential drugs will be discovered in different cancer types by the CR technique.

Traditionally, animal models are common preclinical pharmaceutical tools for toxicity screening. Nevertheless, these models fail to accurately recapitulate the response of human cells to drug toxicity. Thus, human‐derived models are considered ideal platforms for toxicity testing. Because normal CRCs can be cultured in 3D and in vivo models, the CR platform can be used for toxicity assessment. It is established that drug metabolism and detoxification mainly occur in the liver; therefore, the liver is the most susceptible organ to toxic drugs. The commercial primary hepatocytes usually lose their proliferative capacity and liver‐specific functionality in several days of culture. To address this challenge, Su et al successfully established primary human hepatocytes (PHHs) from a range of liver resection materials using CR culture.^129^ As a result, PHHs from young patients could survive for more than 3 months, whereas that from adult patients had a lifespan of 2‐3 months; yet both were more long‐lived than most commercial hepatocytes.^129^ In an in vitro setting, these PHHs maintained proliferative ability, genetic stability, and hepatocyte‐specific functionality at early passages, suggesting that patient‐derived PHHs may serve as valuable models for toxicity testing and liver disease research. Wang et al established human normal limbal epithelial cells from limbal tissues by the CR method.^130^ These CRCs have been identified as novel potential physiological cell models for corneal toxicity assessment. Cytochromes p450 (CYPs) are central in the chemical and drug metabolic process. However, neither transiently cultured primary cells nor immortalized cell lines can maintain high CYPs expression and activity.^62^, ^85^ Zhang and colleagues established human bronchial epithelial cells through CR culturing from three normal bronchial specimens obtained by flexible bronchofiberscopy.^85^ Importantly, these cultured bronchial epithelial cells expressed comparable levels of CYPs as those in lung tissue, and benzo(a)pyrene could induce high expression of CYPs in CRCs.^85^ The kidney is a key organ responsible for the excretion of numerous pharmaceuticals and corresponding metabolites. At present, kidney organoids derived from iPSCs have been established to evaluate the response of renal proximal tubules to nephrotoxic drugs such as cisplatin.^60^ These toxicity tests may also be possible through CR, or combining the two may work better. To summarize, the CR technique can provide a useful in vitro model for drug discovery and toxicity testing.

### 3.5 Regenerative medicine

Regenerative medicine is defined as any biomedical technology that replaces or reconstructs human tissues or organs for therapeutic purposes.^131^ The development of genetic engineering and tissue engineering has greatly accelerated the translation of regenerative medicine to the clinic.^132^, ^133^, ^134^ Considering that normal CRCs can differentiate into origin cells when CR conditions are removed,^79^, ^86^ the CR technology may function well in tissue repair or regenerative medicine.

Currently, stem cell‐based therapies that aim to apply an autologous epithelium to tracheal transplants are in their infancy. This is mainly limited by the current airway epithelial cell culture technology in its scalability, and the inability to culture cells with appropriate differentiation potential and function at clinically relevant time points. Butler et al employed CR culturing and found its ability for the rapid expansion of functional human airway basal cells.^135^ These cells were capable of pluripotent differentiation in vitro and could repopulate tracheal scaffolds in a heterotopic transplantation xenograft model,^135^ suggesting its suitability for use in tracheal reconstruction. Consistently, LaRanger et al reported that CR bronchial epithelial cells could differentiate into the upper airway bronchial epithelium and lower airway alveolar structures after 12 days of implantation into the decellularized mouse lung.^136^ In addition, by CR culturing, primary airway epithelial cells can also be generated from initially cryopreserved biopsy samples.^137^ With this convenience, CR will greatly facilitate the transfer of samples between the clinical facilities and the specialist laboratories and has the potential to transform biobanking repositories.^62^ In addition, CR can be combined with gene editing technology,^138^, ^139^ suggesting the potential of molecular mechanism study and gene therapy.

Within urology, there is a strong demand for regeneration technology, especially with regard to organ transplantation, urinary tract reconstruction, tissue repair, and prosthetic development. For end‐stage renal disease, organ transplantation remains curable therapy. However, given the lack of organs, an increasing number of researchers have attempted to find solutions from regenerative technology. At present, the cellular complexity of the kidney (>20 different types of epithelial cells) still hinders the pace at which researchers can culture patient kidneys in vitro.^13^ Surprisingly, multicellular kidney organoids have been successfully established using human pluripotent stem cells. Within the organoids, individual nephrons consist of proximal and distal tubules, early loops of Henle, and glomeruli which contain podocytes.^60^ Questions remain surrounding the CR technique. Can it efficiently generate different types of functional cells to form an organ‐like entity rather than troubled by the difficulty of differentiation of induced or ESCs?

## 4 CHALLENGES AND FUTURE PERSPECTIVES

In spite of the encouraging progress of CR in cancer research, several challenges within this technique remain to be solved. First, regarding the culture system, CRCs can be contaminated with feeder cells causing interference with subsequent assays.^120^ For this limitation, Palechor‐Ceron et al found that direct physical contact between feeder cells and epithelial cells is not essential for the induction of CR and immortalization.^140^ Hence, the contamination may be avoided by a simplified culture system that replaces feeder cells with a medium that has been conditioned by irradiated feeder cells.^140^

Second, the overgrowth of benign epithelial cells has been reported during malignant CRCs culturing,^83^, ^141^, ^142^, ^143^ which also remains a challenge during cancer organoid derivation.^13^ Generally, this challenge may be attributed to the contamination by normal epithelial cells in the co‐culture system. Therefore, it highlights the need for stringent sampling of tumor materials and genomic analysis to validate the origin of established cultures. Additionally, disease stage, tumor genotype, and culture condition are considered important determinants of culture success of tumor CRCs/organoids.^142^, ^143^ Several studies have employed inactivated human dermal fibroblasts^144^, ^145^ rather than mouse embryonic fibroblasts as feeder layers to facilitate cancer cell expansion and it has also been possible to eliminate the feeder cell component from the standard culture system in certain tumor types (such as pancreatic, colonic, and neuroendocrine cancers).^9^ Studies have also been reported to modify other medium components used in the CR system. For example, removal of Wnt/β‐catenin pathway activators Wnt3a, R‐spondin‐1, as well as the BMP/TGFβ antagonist Noggin from the standard medium can selectively expand colorectal cancer cells rather than normal epithelial cells.^146^ Moreover, the use of specific agents may improve the selectivity of cancer cells, such as Nutlin‐3a, an MDM2 inhibitor that can select for cells harboring *TP53* mutations (which occur in half of all human tumors).^147^

Third, the fidelity of tumor‐derived genetic aberrations and tumor phenotypes still remains a challenge, according to reports in certain cancer types.^142^, ^148^, ^149^ In nasopharyngeal carcinoma, Yu et al reported that only two fifth of the derived tumor CRCs could retain part of the mutant genes detected in their parental samples.^149^ In lung cancer, the tumor CRCs also lacked genetic mutations, which were reported to completely disappear at passage 4 in all samples.^142^ Potentially, all of these CRCs that do not display tumor‐specific alterations and phenotypes are either considered as normal epithelial cells or affected by the overexpansion of nonmalignant cells. In the future, modifications to the current CR culture system may be realized to facilitate the efficiency of malignant CRC derivation and to commit the fidelity of genetic aberrations and tumor phenotypes.

Another limitation of the CR technique is the inhibition of outgrowth of human stromal cells in the origin tissues. Hence, it is difficult to evaluate the influence of stromal cells on tumor cell growth and their effect on the response of the tumor cells to therapeutic agents. This phenomenon is mainly attributed to the effect from J2 feeder cells^9^. This barrier may be overcome with the refinement of CR culturing, such as in combination with 3D culture methods.

In addition, the use of the ROCK inhibitor Y‐27632 may interfere with tumor cell migration and invasion behavior in vitro, as it alters the actin cytoskeleton.^9^ However, this phenomenon needs further investigation.

Last, the use of CR technology in urological cancer research (other fields may be the same) is only in its infancy, especially regarding tumor biology, clinical diagnosis, and tissue regeneration. Even so, the CR technique may preferentially facilitate toxicity testing and regeneration research due to the convenience of normal epithelial cell derivation.

In the future, further validation regarding the reliability of CR technology is necessary. With the refinement of CR and its integration with other advanced models, such as organoid and PDX, the CR technology has great potential to transform biobanking repositories and generate more useful models for cancer research.

## 5 CONCLUSIONS

The CR technique enables the efficient establishment of patient‐derived primary tumor and/or normal cells without any exogenous gene transduction. Removal of feeder cells and ROCK inhibitors allow the CRCs to differentiate normally. Under in vitro culturing, normal CRCs can retain normal karyotypes and differentiative potential, whereas CRCs derived from tumors may retain their tumorigenic phenotypes. All these features enable CR to serve as a promising platform to facilitate urological cancer research, including research on cancer biology, noninvasive diagnosis, high‐throughput drug screening, personalized treatment, and regenerative medicine. Nevertheless, the CR method has several limitations to overcome. In the future, with the refinement of CR and its combination with other advanced models (in vitro/in vivo), the CR technique has great potential to transform biobanking repositories and generate more useful models for cancer research.

## CONFLICT OF INTEREST

The authors declare no conflict of interest.
